# Oriented assembly of invisible probes: towards single mRNA imaging in living cells†
†Electronic supplementary information (ESI) available. See DOI: 10.1039/c5sc04369g


**DOI:** 10.1039/c5sc04369g

**Published:** 2016-02-04

**Authors:** Xiang-Ling Li, Zhuo-Lei Zhang, Wei Zhao, Xing-Hua Xia, Jing-Juan Xu, Hong-Yuan Chen

**Affiliations:** a State Key Laboratory of Analytical Chemistry for Life Science and Collaborative Innovation Center of Chemistry for Life Sciences , School of Chemistry and Chemical Engineering , Nanjing University , Nanjing 210023 , China . Email: weizhao@nju.edu.cn ; Email: xujj@nju.edu.cn

## Abstract

Due to the complexity of biological systems and the ultralow concentration of analytes, improving the signal-to-noise ratio and lowering the limit of detection to allow highly sensitive detection is key to biomolecule analysis, especially intracellular analysis. Here, we present a method for highly sensitive imaging of mRNA in living cells by using novel invisible oriented probes to construct a turn-on signal generation mechanism from zero background. Two DNA probes (S1 and S2) are asymmetrically modified on two small gold nanoparticles (AuNPs) with a diameter of 20 nm. The hybridization of the two DNA probes with a single target mRNA leads to the formation of an AuNP dimer which shows a prominent plasmonic coupling effect. It generates a strong scattering signal from zero-background under a dark-field spectral analysis system. The unique design of the oriented assembly dimer has the ability to easily discriminate the target signal from the inherent cellular background noise in intracellular detection, thus making this approach a valuable technique for imaging single survivin mRNA and monitoring the distribution of survivin mRNA in tumor cells.

## Abstract 2

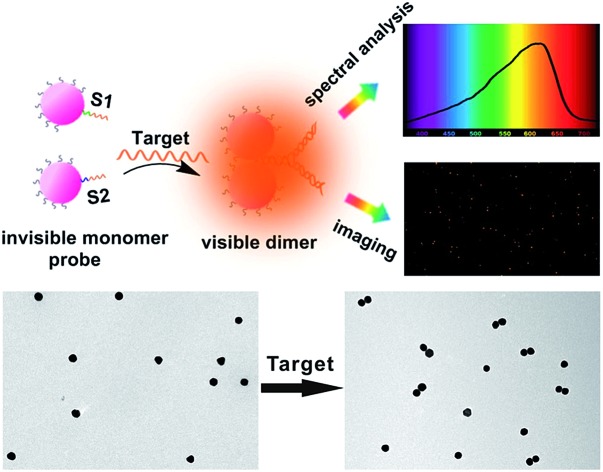
We construct a LSPR sensor by invisible oriented probes with zero background for genetic material sensitive detection in living cells.

## Introduction

Plasmonic engineering of nanomaterials has received great attention since it is capable of manipulating light at nanoscale dimensions. The nanoparticle composition, size, shape, orientation, the distance between the particles and the local dielectric environment determine the plasmon resonance wavelength.^1^–^19^ Jain and co-workers calculated the absorption and scattering properties of gold nanoparticles of different sizes, shapes and compositions.^17^ Zhong *et al.* made theoretical study of the influence from the distance between gold nanoparticles to optical absorbance.^18^ Lan *et al.* provided experimental observations of how scattering intensity increased with decreasing inter-particle distance.^19^ By controlling these parameters, the localized surface plasmon resonance (LSPR) can be predicted and tuned. Recently, lots of work has been carried out to detect biomolecules or follow the dynamical biophysical processes by recording the wavelength shifts of LSPR of noble metal nanoparticles *via* dark-field microscopy (DFM).^20^–^30^ For example, Alivisatos' group monitored the plasmon coupling change of a single plasmonic nanoparticle pair to study the kinetics of single DNA hybridization.^22^ Storhoff's group designed a colorimetric detection strategy for sensitive detection of nucleic acid sequences by monitoring the scattered light change of gold nanoparticles.^30^ These methods show prominent achievements in single molecule level sensing and imaging. However, one common limitation, which can't be ignored, is the close relevance between the particle properties and its LSPR. For instance, large size nanoparticles with stronger plasmon resonance provide simple and visible detection, but offer a strong background signal at the same time.^31^–^33^ In addition, the long-term stability of sensors tends to be inversely proportional to nanoparticle size. Inspired by the theory of tuning LSPR through altering inter-particle distance, we designed a LSPR biosensor with relatively small nanoparticles. In our strategy, 20 nm AuNP monomers with zero LSPR background were introduced as probes, after target binding, visible plasmonic dimers with a much higher scattering efficiency formed, causing a “turn-on” phenomenon.^18^,^19^ In this process, only a single target molecule was required to form a dimer with a significant plasmon resonance coupling effect, which remarkably increased the sensitivity of the LSPR sensor to single-cell level. As far as we know, this is the first time for using an invisible oriented probe to construct a LSPR biosensor with zero background for sensitive detection at single-cell level. The strong optical coupling effect allows us to demonstrate scattering measurements of intracellular analytes as well as ssDNA oligos.

**Scheme 1:**
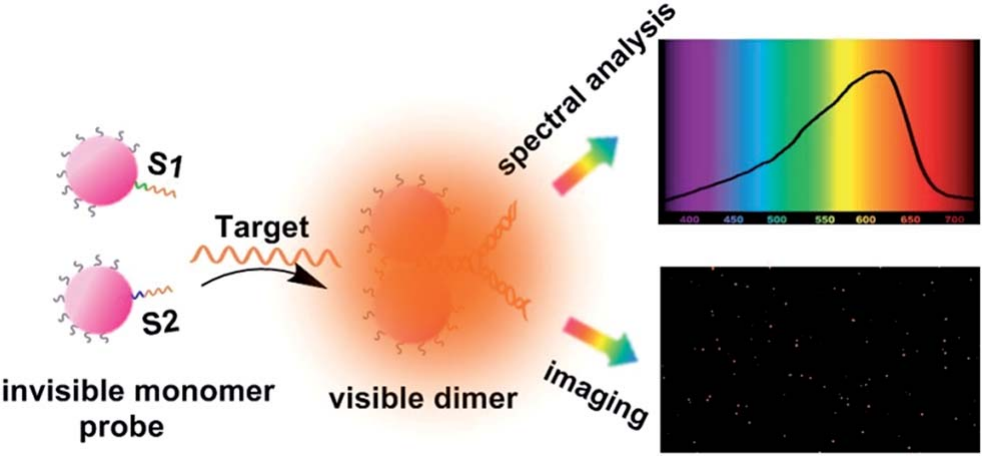
Schematic representation of single mRNA analysis under a dark-field spectral analysis system.

Survivin mRNA, which is responsible for survivin overexpression in all of the most common human malignancies, has become a significant tumor marker.^34^,^35^ Highly sensitive monitoring of survivin mRNA transcripts is of great significance for early disease detection and screening.^36^–^40^ In this study, we used single asymmetrically modified plasmonic AuNPs as independent invisible monomer probes for monitoring genetic material-survivin mRNA in living cells (Scheme 1). Specifically, two batches of asymmetric PEGylated AuNPs (due to our instrument signal-to-noise ratio, 20 nm AuNPs were chosen) were functionalized with two thiolated DNA probes (designated as S1 and S2). Each probe consists of two sections: one is the complementary strand for the other probe, and the other one is complementary to target mRNA sequences. Details are described in the methods section. After modification, these nanoparticles served as invisible monomer probes. When a complementary survivin mRNA was introduced to the monomer probes, their oriented assembly led to the formation of a Y-shaped DNA origami-induced AuNP dimer. The dimer formation induced a significant plasmon resonance coupling effect, resulting in a noticeable scattering signal.

## Results and discussion

### Probe characterization

**Fig. 1:**
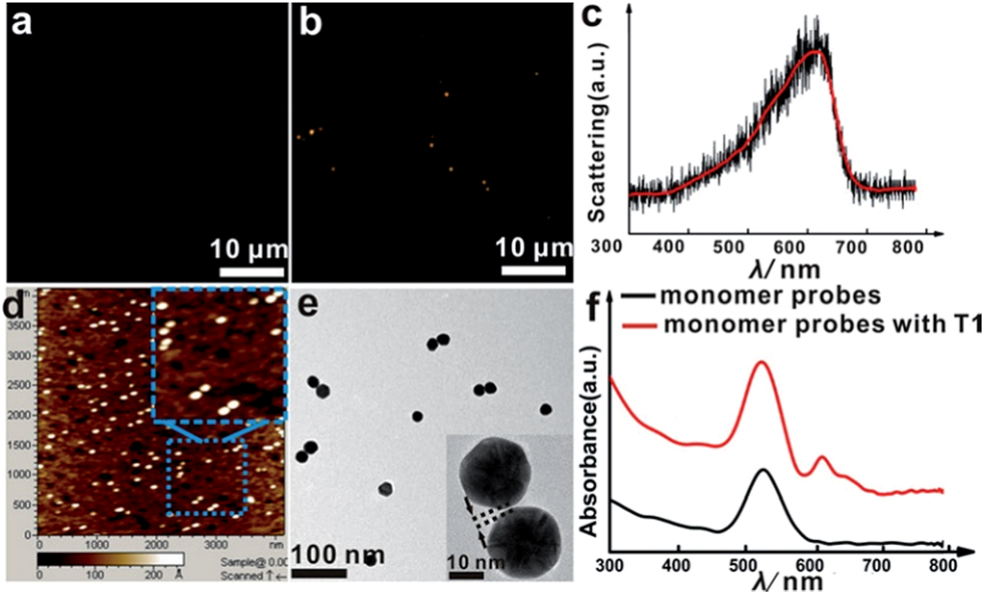
Dark-field images of the monomer probes (AuNP-PEG-S1 and AuNP-PEG-S2) on a glass slide before (a) and after (b) the addition of target molecules (T1, 0.01 pM) at 37 °C for 2 h. (c) Typical scattering spectrum of a single dimer *in vitro*. (d) AFM image of the sample after adding the target. (e) TEM characterization of the reaction solution after adding the target at 37 °C for 2 h, the inset is a HRTEM image of a dimer. (f) Optical absorption spectra of the asymmetrically functionalized monomer probe solution before (black curve) and after addition of target DNA (T1, 10 pM) at 37 °C for 2 h (red curve).

The asymmetrical modification process of AuNPs was characterized by zeta potential analysis (Fig. S1†). Additionally, the concentration of the obtained AuNP-PEG could be accurately calculated by UV-Vis absorbance measurements (Fig. S2†). Based on theoretical calculations and fluorescence analysis, each AuNP monomer was calculated to carry roughly 32 PEG molecules and 5 ± 1 thiol-oligonucleotides (Fig. S3 and S4†). Fig. 1a shows that the monomer probes give a zero background in dark-field detection. The scanning electron microscope (SEM) images indicate that in the absence of the target sequence, the asymmetrically modified monomer probes are dispersed without spontaneous aggregation in the *x*–*y* plane (Fig. S5†). When the target (synthesized oligonucleotides, T1) was introduced and reacted with the monomer probes at 37 °C for 2 h, an obvious invisible to visible transition (orange spots) and a typical dimer scattering could be clearly observed due to strong plasmonic coupling (Fig. 1b and c). A contrast experiment was performed to verify the distinction of scattering spectra between a monomer and dimer. Since the scattering intensity of a single 20 nm Au NP was too weak to measure, we chose a 30 nm Au NP as the probe. The results revealed that the scattering peak of a dimer centres at 610 nm, which is red-shifted compared to that of a monomer (540 nm). Meanwhile, the scattering intensity of a dimer is nearly 6-fold that of a monomer. Besides, we utilized the finite-difference time domain (FDTD) method to simulate the LSPR properties of gold monomers and dimers with different diameters. The theoretical simulation showed that no matter if choosing a 20 nm or 30 nm Au NP mode, the LSPR spectrum of the dimer was distinctly red-shifted compared to that of the monomer, which was highly consistent with the experiment results. Detailed information is provided in the supporting section (Fig. S6 and S7†). These results were further confirmed by atomic force microscopy (AFM) and transmission electron microscopy (TEM) characterization (Fig. 1d and e). High resolution transmission electron microscopy (HRTEM) showed that the average interparticle distance of the dimer was less than 2 nm, which agrees well with other reports.^41^,^42^ As the plasmonic coupling effect of two noble metal nanoparticles is distance-dependent, the plasmonic coupling effect is gradually increased with the decreasing of interparticle distance.^19^,^22^ Hence, the results of the UV-Vis absorption measurements also confirmed the remarkable plasmonic coupling effect of the dimers. As shown in Fig. 1f, a specific spectral peak for the dimers at 608 nm was detected, in addition to the monomer absorbance peak at 527 nm, after introducing the target to the monomer probes, indicating that the oriently formed dimer has a strong plasmonic coupling effect.

**Fig. 2:**
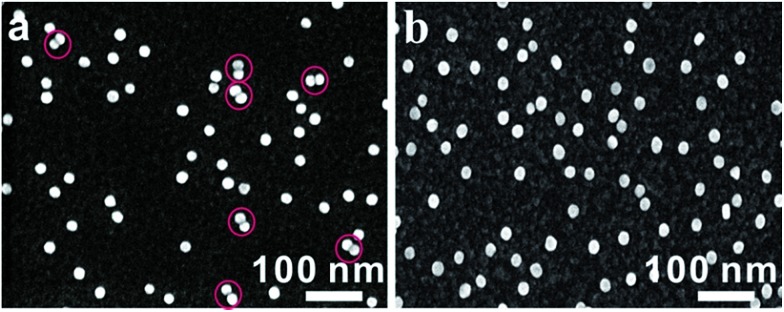
SEM images of asymmetrically modified monomer probes incubated with the target strand (T1, 10 pM, (a)) and with the control strand (M-C1, 10 pM, (b)) at 37 °C for 2 h.

For most intracellular detection, it is necessary to construct plasmonic probes that have the ability to distinguish very closely related sequences such as a single-base mismatch. To accomplish this, single-mismatched oligonucleotides (M-C1) were used in the control experiment for investigating the selectivity of the monomer probes. When the target (T1) was added, several dimers were observed (Fig. 2a). On the contrary, no dimers were detected in the control experiment (Fig. 2b), demonstrating that there was no hybridization reaction inducing the formation of dimers. All of these results revealed the excellent selectivity of this strategy.

As DNA hybridization and temperature are closely related, a melting temperature assay was performed to provide a clear understanding of the above results. Interestingly, we observed a significant difference in the melting temperature (*T*_m_) between the target and control oligonucleotides (Fig. S8†). The melting temperature (*T*_m_) for the perfectly matched oligonucleotides (T1) was much higher than that of the control oligonucleotides. This was attributed to the instability of the DNA duplex formed by control oligonucleotides (M-C1 or H-C1) and monomer probes. Additionally, in the melting temperature test, we found that while reaching the melting temperature, the duplex formed by T1 and monomer probes unwound, which caused the dimer spectral peak to disappear and only the absorption peak of the monomer probe was retained. This further confirmed the formation of dimers upon target hybridization.

The selectivity of the monomer probe was further investigated by a dark-field assay. In the presence of the target, more and more orange spots, representing the scattering of AuNP dimers, appeared with prolonging the incubating time (Fig. S9a†). While in the presence of the control oligonucleotide (M-C1), no signal was detected, even up to 2 h (Fig. S9b†). All of these results revealed that, based on the specific merit of this approach, the monomer probes have outstanding selectivity for intracellular imaging analysis.

Excellent stability of a detection probe is essential in intracellular analysis and biological studies. Before investigating the performance of the plasmonic probes in biological solutions, we first studied the stability of the monomer probes in water, PBS buffer solution, cell medium and serum. As shown in Fig. S10a, † the asymmetrically modified monomer probes exhibited excellent stability in all biological solutions. The UV-vis optical absorption spectra of the monomer probes dispersed in different solutions just showed a sharp peak at ∼530 nm, and no broad extinction peak caused by a large aggregation of probes was detected (Fig. S10b†). Meanwhile, the process was also characterized by dynamic light scattering (DLS) measurements. DLS measurements showed that the size distribution of the asymmetrically modified monomer probes in water, PBS, cell medium and serum (Fig. S10c–f†), was mostly within a narrow range from 25 nm to 35 nm. All of these results revealed that the monomer probes are soluble in water and do not aggregate in salts or protein-rich solutions such as cell medium and serum, indicating that after asymmetrical modification, the monomer probes have excellent stability in biological solutions and are robust candidates for intracellular detection.

**Fig. 3:**
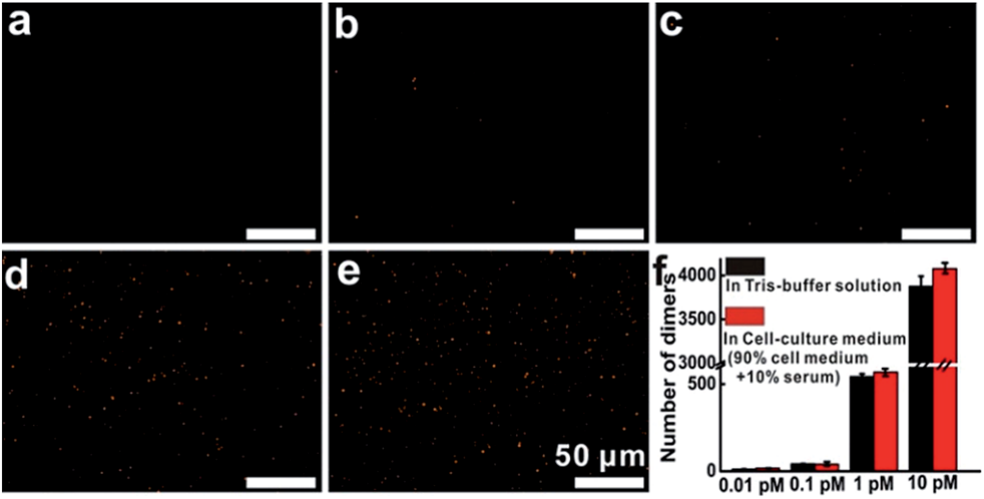
Dark-field images of nanoparticles after the addition of different concentrations of target oligonucleotides (T1), from (a to e) in cell-culture medium: 0, 0.01, 0.1, 1, 10 pM, each image area is 235 μm × 176 μm. (f) The number of dimers at different concentration points.

Then, additional experiments were performed to evaluate the target detection in Tris-buffer solution or cell-culture medium. The results indicated that in Tris-buffer solution, a higher concentration of the target would lead to a larger number of dimers forming and almost 4000 dimers (analysis range, 235 μm × 176 μm) could be observed with a target concentration of up to 10 pM (Fig. S11†). As expected, the trend of dimer formation based on target hybridization in cell-culture medium was consistent with that in Tris-buffer solution (Fig. 3). In the dark-field detection, the number of orange spots representing dimer scattering increased monotonically with increasing the target concentration in cell-culture medium, indicating that the target-induced formation of nanoparticle dimers have excellent performance in complex media. All of these results revealed that target hybridization enabled two monomers to assemble by a single target mRNA to form a dimer, yielding plasmonic coupling and a turn-on signal from zero background. These significant improvements in stability, selectivity and sensitivity demonstrate that this technique has the capability of imaging and monitoring mRNA in complex cellular microenvironments.

**Fig. 4:**
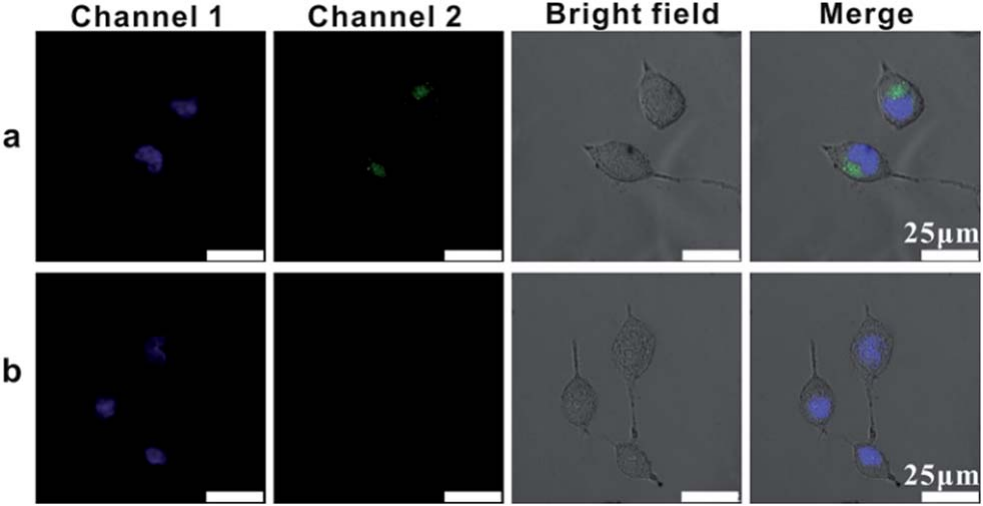
Confocal fluorescence images of HeLa cells after incubation with the location probes for 4 h at 37 °C (a) and 4 °C (b). The images from left to right: Channel 1 represents cells stained by Hoechst 33342, Channel 2 represents fluorescence intensity of AlexaFluor 488 in cells treated with location probes, bright image and merged images, respectively.

In order to image single mRNA in cancer cells, we investigated the intracellular uptake pathway and the intracellular localization of AuNP probes in mammalian cells. Location probes, used for confirming the exact locations of probes in cells, were modified with AlexaFluor 488 labelled oligonucleotide (L1) instead of probes S1 and S2. Human cervical cancer (HeLa) cells were incubated with the location probes at 4 °C or 37 °C. After the transfection process, the cells were then stained with the DNA-specific dye bis-benzimidazole Hoechst 33342 at 37 °C for 10 mins to highlight the cell nucleus region (Channel 1, Fig. 4).^43^ While incubated with the probes at 37 °C, a large amount of green spots emitted from the location probes in the cells was observed (Channel 2, Fig. 4a), confirming that vast probes could be ingested by the cells. Nevertheless, almost no fluorescence from the location probes could be observed in the cells incubated at 4 °C (Fig. 4b), suggesting that few probes could be internalized by the cells at this temperature. All of these results demonstrated the efficient uptake of probes at 37 °C and that the uptake mechanism of this type of probe is an energy-dependent endocytosis.

**Fig. 5:**
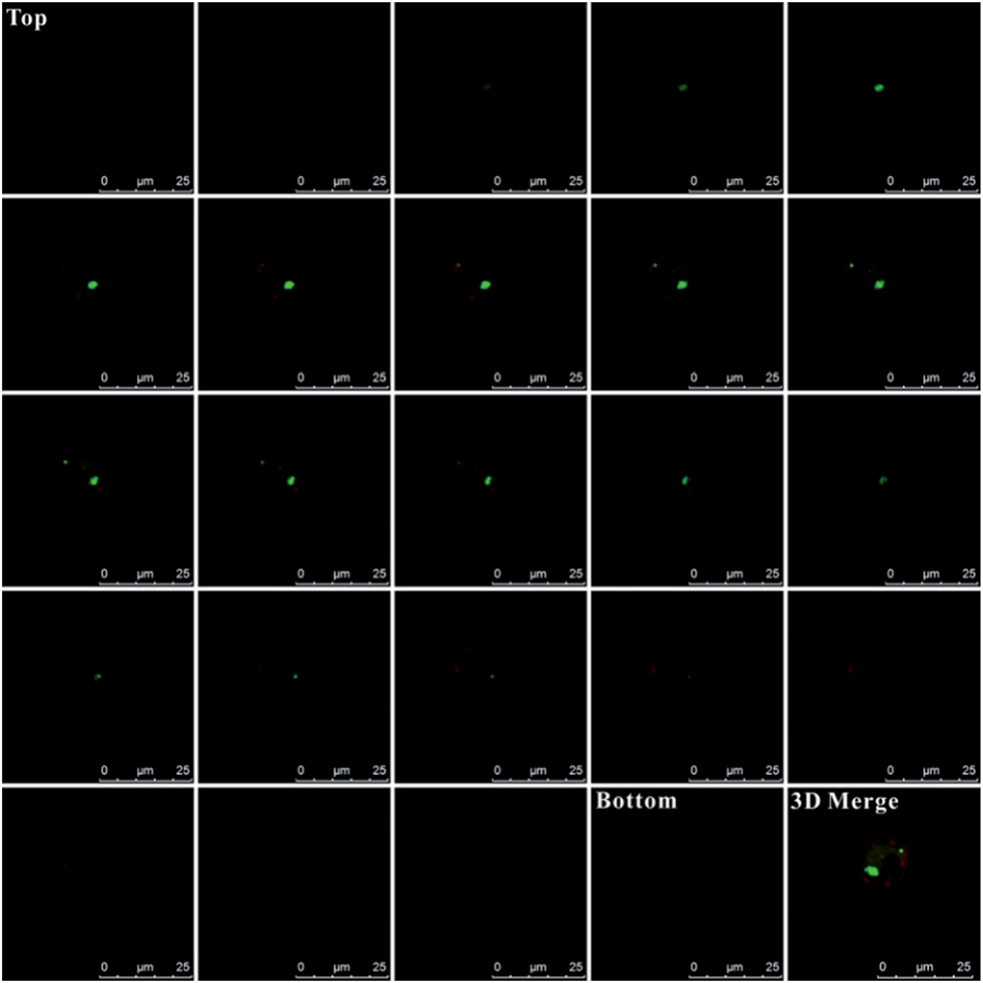
Z-stack images of composite emissions from AlexaFluor 488 (green fluorescence, presenting the probes location) and plasma membrane stain (red fluorescence, presenting cell membrane location). The images were taken in a series of 24-step (0.5 μm-step-sizes) measurements, starting from above the cell (top) toward the bottom of the cell.

Additionally, for affirming the location of probes in the cells, Z-stack analysis of the target cells was performed by confocal microscopy. As presented in Fig. 5, from the top toward the bottom of the cells, the red fluorescence emitted by the plasma membrane stain became intense and displayed ring-like features along the membrane peripheries while focusing on the middle. The emission fluorescence from AlexaFluor 488 (green fluorescence, presenting the probes location) became more intense toward the center of the cell. These features strongly indicated that the probes were inside the cells instead of being adsorbed on the membranes, which would otherwise display a ring-like overlap with the membrane, especially at middle *z* positions. Additionally, as shown in the 3D merge image and the Video S-1,† the green colors inside the cells also indicate that these probes were internalized by cells and were inside the cells instead of being adsorbed on membranes.

### Survivin mRNA imaging in living cells

**Fig. 6:**
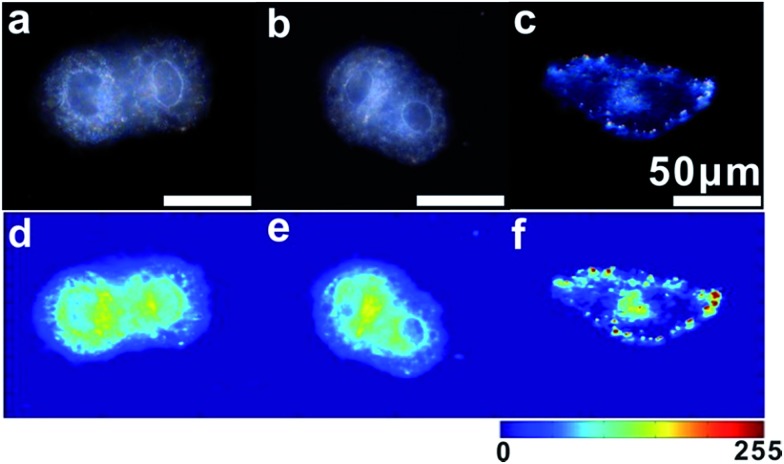
Dark-field images of HeLa cells (a), cells incubated with control probes (b) and monomer probes (c). The corresponding scattering intensity of these samples described by a pseudo-color map (from d to f).

For imaging single mRNA in living cells, a control experiment was first used to investigate whether the monomer probe could be used for constructing relative zero-background for intracellular analysis. The control probes used in the control test were modified with random probes (R1 and R2) instead of probes S1 and S2, which were noncomplementary to the target mRNA. As survivin mRNA is overexpressed in human cervical cancer (HeLa) cells, HeLa cells were used in intracellular analysis.^44^ HeLa cells incubated with control probes or monomer probes were observed using dark-field microscopy. Due to the complexity of intracellular environments,^45^ cells themselves have an intense scattering signal (Fig. 6a). After incubation with the control probes, no scattering signal from the monomer control probes was observed (Fig. 6b), which was attributed to the scattering of the small probes being too weak to be detected. In addition to this, no dimer or scattering signal was detected in these cells. For cells incubated with the monomer probes, the final result was completely different. The scattering signals from the dimers were highly intense compared with that of the cell (Fig. 6c), which made it easy to discriminate specific target signals from the intracellular environment background noise. These results revealed that the monomer probes have the capacity of constructing relative zero-background and can sensitively monitor single mRNA in living cells. In addition, a cell viability assay was performed to analyze the biocompatibility and toxicity of the asymmetrically modified probe. The results revealed that the probe exhibited negligible effects on growth in HeLa cells (Fig. S12†), and the advantages of the probes shown here made it an excellent candidate in cellular detection.

**Fig. 7:**
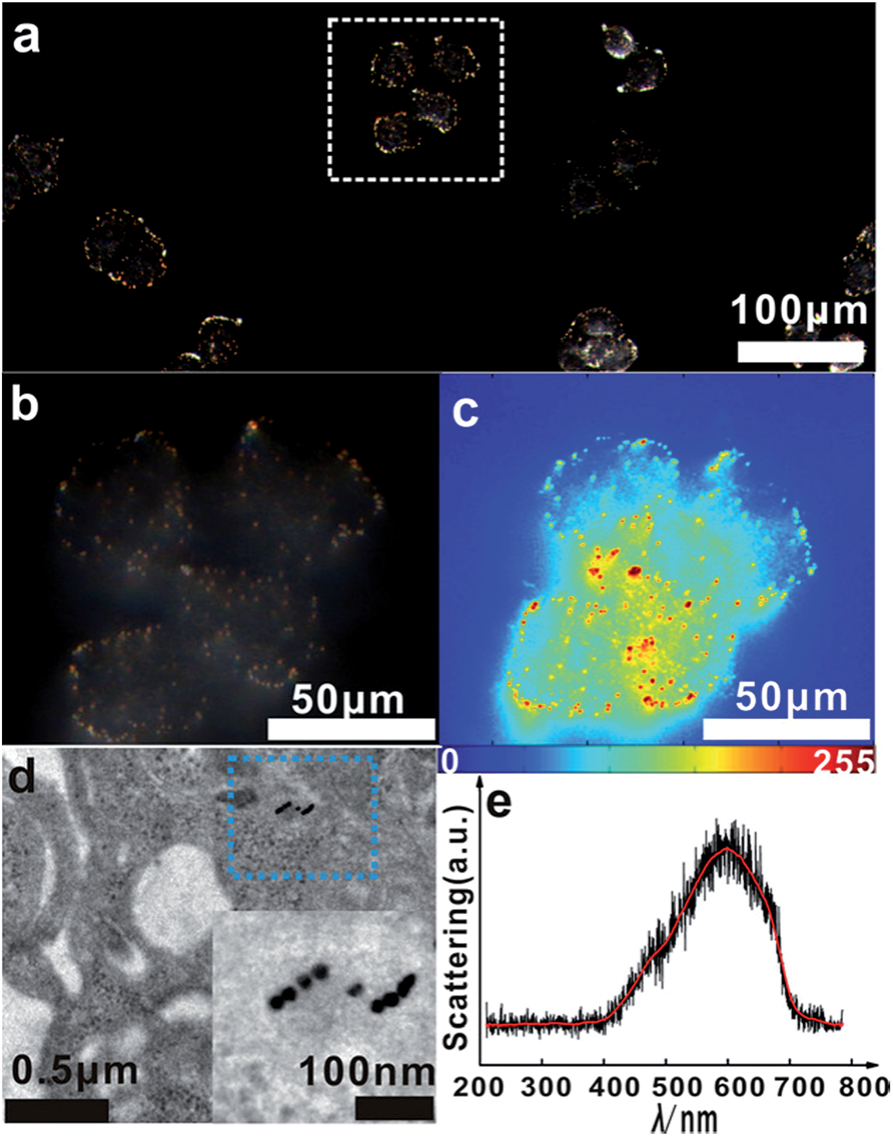
(a) Dark-field images of HeLa cells incubated with monomer probes for 4 h. (b) The magnified dark-field image of the white box in (a), the magnifying power is 37.8×. (c) The corresponding scattering intensity of these cells described by a pseudo-color map. (d) TEM image of HeLa cells incubated with monomer probes for 4 h, the inset is the magnified image of the blue box. (e) Representative scattering spectrum of a dimer in a living HeLa cell.

Based on the above finding, we next investigated the capabilities of the monomer probe for detecting and imaging single mRNA under intracellular conditions. After incubation with the monomer probes for a period of time, some bright orange spots representing a dimer scattering signal were found in the cytoplasm of the cells, indicating that two monomers successfully assembled to a single survivin mRNA and formed a dimer (Fig. 7a). In the magnified region of the image shown in Fig. 7a (white box), dimer scattering signal-specific orange spots were clearly observed in a single cell, demonstrating that the genetic material-survivin mRNA could be directly mapped *in situ* by the Y-shape DNA origami-induced AuNP dimers (Fig. 7b). As shown in the scattering intensity mapping of the whole cell, the dimer signal was much stronger than the background noise arising from the intracellular microenvironments and could be easily discriminated from this background for intracellular analysis (Fig. 7c). During TEM analysis, several dimers were found in the cytoplasm of HeLa cells after incubation with monomer probes (Fig. 7d), which revealed that the hybridization of the two DNA probes with single target mRNA can lead to the formation of a dimer and achieve imaging analysis of mRNA *in situ*. The characteristic scattering spectrum of single dimer at ∼610 nm was detected in the cellular imaging by spectral analysis (Fig. 7e). These results demonstrated that the LSPR monomer probes have tremendous merits for monitoring single mRNA in living cells due to the scattering signals found in the cytoplasm of the cells.

**Fig. 8:**
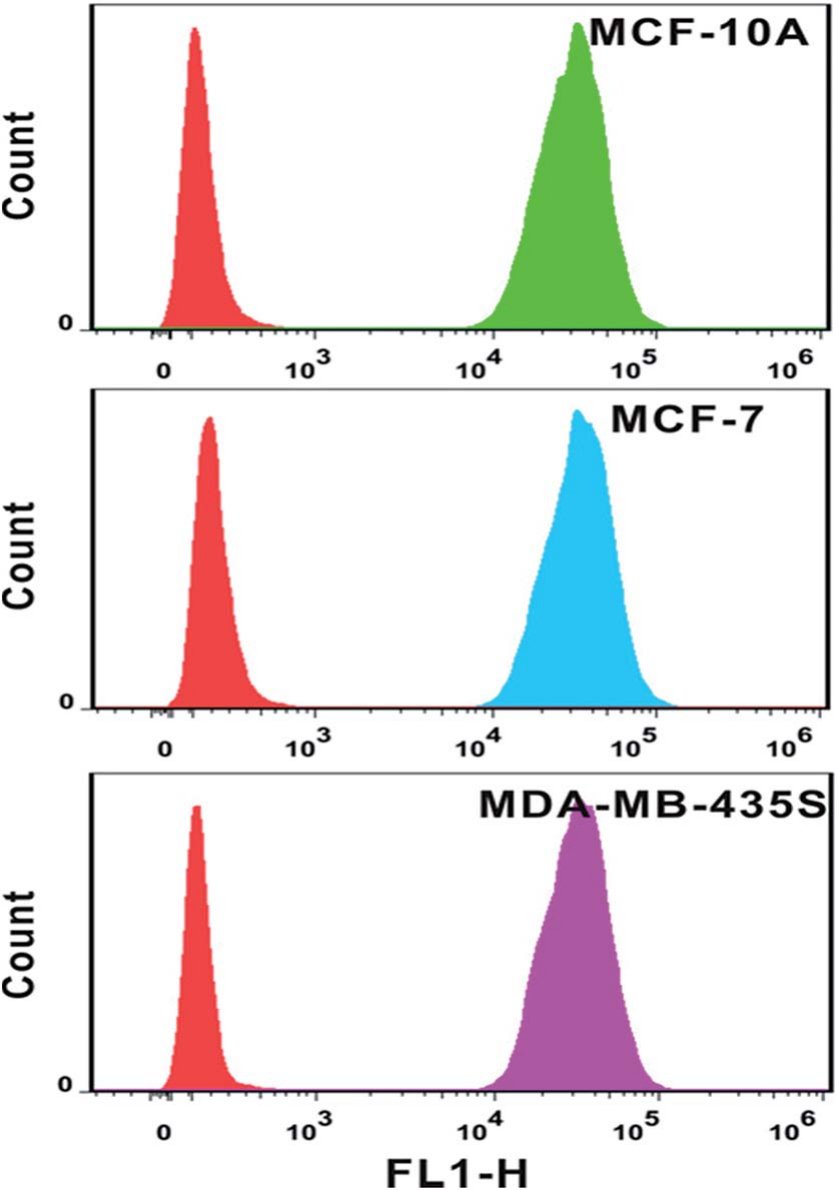
Flow cytometry results of MCF-10A cells, MCF-7 cells and MDA-MB-435S cells after incubation with location probes for 4 h at 37 °C. Lines from left to right: cell control; cell incubated with probes. FL1-H represents FITC signal.

The analysis of mRNA levels *in situ* is of great significance for early disease detection and biological studies, which can be further used to facilitate the gene expression for selection of initial therapy and monitoring of treatment progress. Hence, we investigated whether this plasmonic approach can be applied for determining variations in the presence and quantities of survivin mRNA in different cell lines. Firstly, flow cytometry analysis was used to determine the amount of probes in each cell line. Based on the analysis of the intensity of fluorescence from quantification probes, we can clearly find that the amount of probes in the normal immortalized human mammary epithelial cells (MCF-10A), human breast adenocarcinoma cells (MCF-7) and metastatic human breast cancer cells (MDA-MB-435S) was not largely different and located on the same order of magnitude (Fig. 8).

**Fig. 9:**
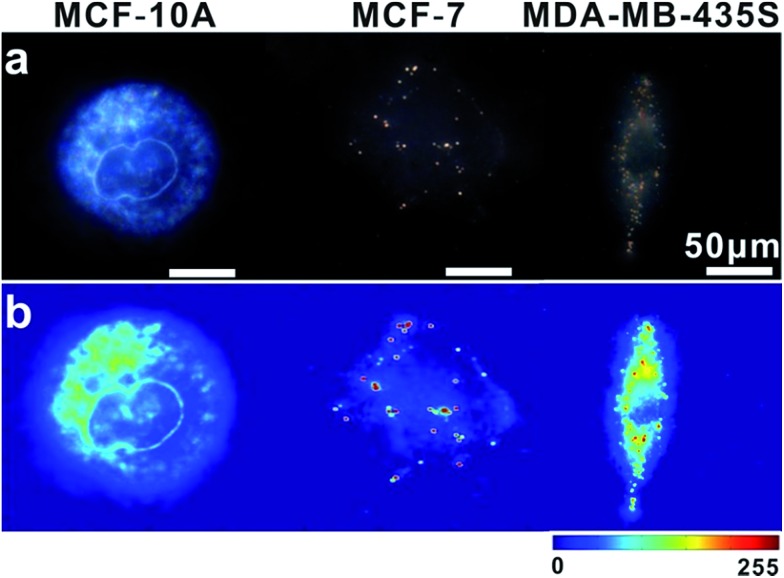
(a) Dark-field images of MCF-10A, MCF-7 and MDA-MB-435S cells incubated with monomer probes. (b) The corresponding scattering intensity of these samples described by a pseudo-color map.

In intracellular detection, no dimer signal was detected in MCF-10A cells. The dimer scattering signal was detected in both MCF-7 and MDA-MB-435S cell lines (Fig. 9). Importantly, based on counting the number of dimers, we found that nearly 71 ± 4 dimers per cell were detected in the MDA-MB-435S cell line, while 19 ± 2 dimers per cell were detected in the MCF-7 cell line. When analysing HeLa cells, roughly 38 ± 3 dimers per cell were detected (Fig. 8). This result was further verified by real-time quantitative, reverse-transcription PCR (qRT-PCR) analysis, which showed a moderate level of survivin mRNA in MCF-7 cells, a high level in HeLa cells and an extremely high level in MDA-MB-435S cells (Fig. S13†). The results revealed that the tendency of survivin mRNA expression level in various cell lines detected by the dark-field analysis system was consistent with the results detected by qRT-PCR analysis. All of these results indicated this LSPR sensor can achieve single mRNA imaging *in situ* and monitor its expression level in various living cells. Considering the outstanding performance of the oriently assembled plasmonic dimer, it may find future use in bimolecular interaction, monitoring and tracking biomolecules at a single living cell level.

## Conclusion

In this study, we present a smart single mRNA imaging approach in living cells based on target-induced formation of nanoparticle dimers. The hybridization of these invisible probes to a single target mRNA, which resulted in an obvious “turn-on” signal from zero-background, makes it a highly sensitive LSPR sensor. The Y-shaped DNA duplex actuation of nanoparticle dimers, which can greatly minimize the interparticle distance while maximizing the plasmon coupling effect, is strong enough for imaging single mRNA in living cells. With proper linker functionalization, such a platform could be extended and serve as a general basis to pave a way for imaging various biomolecules as well as mRNA in living cells.

## Experimental

### Preparation of asymmetrically modified Au nanoparticles (NPs)

AuNPs with an average diameter of 20 nm were purchased from Ted Pella, Inc. Amine group coated glass slides served as substrates for the adsorption of tannic acid capped AuNPs based on electrostatic attraction. The process of slide silanization was carried out according to the method of Yoon and co-workers.^46^ Glass slides (25 mm × 9 mm) were first cleaned in a “piranha” solution (30% H_2_O_2_ mixed in 1 : 3 ratio with concentrated H_2_SO_4_) for 1 h. After cooling to room temperature, these glass slides were washed thoroughly with distilled water, and dried with nitrogen. Then, these clean glass slides were immersed in an ethanolic solution of (3-aminopropyl)triethoxysilane (APTES, 1% v/v) for 30 min for aminosilanization. The APTES-modified glass was washed with ethanol and distilled water, and dried in an oven at 120 °C for 3 h. The silanized glasses were immersed in an aqueous solution of tannic acid-capped AuNPs for 4 h, and then they were quickly rinsed with water and placed in a solution of 1 mM SH-PEG-COOH overnight. After being washed and dried, these PEGylated AuNPs were removed from the glasses *via* ultrasonic treatment in 1.0 mL water for 5 min. The nanoparticles were then centrifuged (8000 rpm, 10 min) and washed three times with distilled water, then resuspended in 1.0 mL distilled water for the next modification or UV-vis absorption measurements.

Two batches of PEGylated AuNPs were functionalized with thiolated oligonucleotides according to ref. 47 with a slight modification. Briefly, in order to reduce disulfide bonds, the thiol oligonucleotides (S1, S2, 0.1 mM) were deprotected in Tris–HCl buffer (20 mM, 0.1 M NaCl, pH 7.4) with 0.1 mM TCEP at room temperature for 1 h. Then, the deprotected oligonucleotides (S1 or S2, 5 μL, 0.18 μM) were added into 500 μL asymmetrically PEGylated AuNPs. After 24 h incubation, the NPs were centrifuged (8000 rpm, 10 min) and washed three times with Tris–HCl buffer, and resuspended in buffer solution (100 μL) to obtain the asymmetrically modified monomer probes. The control probes for intracellular experiments were formed by the above procedure using random probes (R1 and R2) instead of S1 and S2.

The location probes for probe location and quantitative analysis in intracellular detection were synthesized by the following procedure. Briefly, the thiol oligonucleotides (L1, 0.1 mM) were deprotected in Tris–HCl buffer (20 mM, 0.1 M NaCl, pH 7.4) with 0.1 mM TCEP at room temperature for 1 h. Then, the deprotected oligonucleotides (L1, 5 μL, 0.18 μM) were added into 500 μL asymmetrically PEGylated AuNPs. After 24 h incubation, the NPs were centrifuged (8000 rpm, 10 min) and washed three times with Tris–HCl buffer, and resuspended in buffer solution (100 μL). In order to avoid the fluorescence of AlexaFluor 488 being quenched by the gold particle, the probe L2 was used to hybridize with L1, which would induce the fluorophore far away from the quencher surface and thus restore the fluorescence.^43^ Specifically, after the asymmetrical modification, the thiol oligonucleotide solution (L2, 5 μL, 0.18 μM) was added to the above buffer solution containing the asymmetrically modified probes. After incubation at 37 °C for 2 h, the NPs were centrifuged (8000 rpm, 10 min) and washed three times with Tris–HCl buffer, and resuspended in buffer solution (100 μL) to obtain the location probes. These probes were stored at 4 °C for future experiments. Besides, the quantification probes for the quantitative analysis of probes in intracellular experiments were formed by the above procedure using quantification probes oligonucleotides (Q1) instead of L1.

### Quantitation of oligonucleotides loaded on the AuNP monomer

The thiol oligonucleotides (Q1, 3 μL, 2 μM) were added into 300 μL asymmetrically PEGylated AuNPs (200 pM). After 24 h incubation, the modified monomer probes and redundant oligonucleotides were separated *via* centrifugation, and the fluorescence intensity of the supernate was detected on a fluorescence spectrometer.

### Detection of dimer hybridization *in vitro*

Two batches of monomer probes (modified S1 or S2, 30 μL), at the same concentration of 90 pM, were mixed as the probe solution. After the target (synthetic survivin mRNA, T1) with different concentrations was added to the probe solution, the solution was heated at 65 °C for 2 min, followed by hybridization at 37 °C for 2 h. Dark-field microscopy, SEM and TEM were used to investigate the dimer hybridization in the samples.

### The melting temperature assay of DNA-bound AuNP dimers

3 μL of 10 nM target oligonucleotide (T1) or single mismatched strands (M-C1 or H-C1) was added to 300 μL solution with 900 pM AuNP monomer probes. After hybridization, the absorbance intensity of the AuNP dimer at 608 nm was measured on a microplate spectrophotometer (Epoch 2, BioTek Instruments) at different temperatures. In the measurement process, 1 °C was set as the step and each temperature setting was held for 5 min, then the absorbance intensity was recorded.

### Optical microscopy and spectroscopy

Optical microscopy was carried out on an inverted microscope (IX71, Olympus) equipped with a dark-field condenser (0.8 < NA < 0.92), 60× objective (NA 0.7), a 100 W halogen lamp and a true-color digital camera (DP80, Olympus). The scattering spectra were acquired on a monochromator (Acton SP2358) equipped with an excelon EMCCD (400BR, Princeton) and a grating (grating density: 300 L mm^–1^; blazed wavelength: 500 nm). In this work, the scattering spectra of the dimers were corrected by subtracting the background spectra generated by the instrument itself.

### Cell culture and intracellular survivin mRNA imaging

Human cervical cancer (HeLa) cells and the normal immortalized human mammary epithelial (MCF-10A) cells were cultured in DMEM, metastatic human breast cancer (MDA-MB-435S) cells were cultured in L-15 and human breast adenocarcinoma (MCF-7) cells in RPMI 1640. All cultured media were supplemented with 10% FBS and 100 IU mL^–1^ penicillin–streptomycin. The cells were maintained at 37 °C in a humidified atmosphere (95% air and 5% CO_2_). Cells were seeded on a clean glass slide in a 6-well plate. After 12 h of plating, the culture media was exchanged with fresh serum free basal media (2 mL) containing sodium acrylate (20 μL, 5 M) and the asymmetrically modified monomer probes (200 μL, 50 pM).^48^ After 2 h, the cells were washed with PBS three times and then incubated with fresh culture media for another 2 h. After the above incubation, the cells were washed with PBS three times and then imaged using a dark field microscope or confocal fluorescence microscopy.

### Real-time quantitative, reverse transcription PCR (qRT-PCR)

Total RNA was extracted and purified with the TRLzol Plus RNA Purification Kit (Invitrogen). qRT-PCR was performed using the SuperScript III Platinum SYBR Green One-Step qPCR Kit (Invitrogen) and carried out on a Bio-Rad C1000 Real-Time PCR Detection System (Bio-Rad Laboratories, Inc). The expression levels of the survivin gene were normalized to actin mRNA, an off-target gene. The standard deviation for each data was calculated from three independent experiments. The primer pairs for the survivin gene were survivin forward 5′-ATG GGT GCC CCG ACG TTG-3′, and survivin reverse, 5′-AGA GGC CTC AAT CCA TGG-3′. The primer pairs for the actin gene were actin forward, 5′-ATC ATT GCT CCA CCA GAA CG-3, and actin reverse, 5′-AAG GTA GAT AGA GAA GCC AAG-3′.

### Cell viability assay

Firstly, HeLa cells were seeded in 96-well plates (3000 cells per well) and maintained at 37 °C in a humidified atmosphere (95% air and 5% CO_2_) for 24 h. After 24 h incubation, cells were washed with PBS and then cultured in a medium containing AuNPs or asymmetrically functionalized AuNP-PEG with various concentrations. After 4 h, the cells were washed with PBS and incubated in a fresh medium for an additional 24 h. Then, according to the manufacturer's instructions, 50 μL MTT solution was added to each well and incubated for 4 h at 37 °C. After removing the medium, 150 μL DMSO was added to solubilize the blue-colored tetrazolium, the plates were gently shaken for 5 min, and the optical density values at a 550 nm wavelength were monitored using Thermo Scientific Varioskan Flash. The cell viability was set as 100% in control cells.

## Supplementary Material

Supplementary information

Supplementary movie
