# Supplementary material for: Oriented assembly of invisible probes: towards single mRNA imaging in living cells
Source: Chem Sci. 2016 Feb 4;7(5):3256–63. doi: 10.1039/c5sc04369g (PMC6006471; doi:10.1039/c5sc04369g)
Supplement: Supplementary file 1 [file SC-007-C5SC04369G-s001.pdf]

Electronic Supplementary Material (ESI) for Chemical Science.
This journal is © The Royal Society of Chemistry 2016

Supporting Information

Oriented Assembly of Invisible Probes: Towards Single mRNA Imaging in Living Cells

Xiang-Ling Li, Zhuo-Lei Zhang, Wei Zhao*, Xing-Hua Xia, Jing-Juan Xu*, Hong-Yuan Chen

State Key Laboratory of Analytical Chemistry for Life Science and Collaborative Innovation Center of Chemistry for Life Sciences, School of Chemistry and Chemical Engineering, Nanjing University, Nanjing 210023, China.

*Corresponding author: Email: weizhao@nju.edu.cn, xujj@nju.edu.cn, Tel: +86-25-89687294, fax: +86-25-89687294.

Experimental section

Reagents and Materials. Dulbecco's Modified Eagles Medium (DMEM), Roswell Park Memorial Institute 1640 (RPMI-1640), Leibovitz Medium (L-15), fetal bovine serum (FBS) and trypsin were purchased from Invitrogen. Sodium acrylate, (3-aminopropyl)triethoxysilane (APTES, 99%), dimethyl sulfoxide (DMSO), Tris(2-carboxyethyl)phosphine (TCEP) and O-(3-Carboxypropyl)-O′ -[2-(3-mercaptopropionylamino)ethyl]-polyethylene glycol (Mw 3000, SH-PEG-COOH) were purchased from Sigma-Aldrich. Human cervical cancer (HeLa) cells, human breast adenocarcinoma (MCF-7) cell, cell cytotoxicity assay kit and Hoechst 33342 staining kit were purchased from Key GEN Biotech. The normal immortalized human mammary epithelial (MCF-10A) cell and metastatic human breast cancer (MDA-MB-435S) cell were purchased from Bioleaf Biotech. AuNPs with an average diameter of 20 nm and 30 nm were purchased from Ted Pella, Inc. All other reagents were of analytical reagent grade. The water from Milli-Q (Millipore, Inc., Bedford, MA) was RNase-free by pretreated with diethylpyrocarbonate. The oligonucleotides were synthesized by TaKaRa Bio Inc. (Dalian China) and Shanghai Sangon Biological Engineering Technology & Services Co. (Shanghai China). Detailed DNA sequences and modifications were shown in Table S-1.

Table S-1 DNA sequences	
DNA for in vitro tests	Sequences (5′ to 3′)
chemical synthesized target strand T1[a]	CAAGGAGCTGGAAGGCTGGG
single mismatched strand at middle M-C1 [b]	CAAGGAGCTG C AAGGCTGGG
single mismatched strand at head H-C1 [b]	G AAGGAGCTGGAAGGCTGGG
Monomer probe	
probe S1[c]	SH-$(CH_2)_6$-*CTGTTACTG*CAGCTCCTTG
probe S2	CCCAGCCTTC*CAGTAACAG*-$(CH_2)_6$-SH
Control probe	
random probe R1	SH-$(CH_2)_6$-*CTGTTACTG*CAGCTCTCGT
random probe R2	GGACAGGGCT*CAGTAACAG*-$(CH_2)_6$-SH
Location probe	
probe L1	SH-$(CH_2)_6$-CTGTTACTGCAGCTCCTTG-AlexaFluor488
probe L2	CAAGGAGCTGCAGTAACAG
Quantification probe	
probe Q1[d]	SH-$(CH_2)_6$-CTGTTACTGCAGCTCCTTG-FITC
qRT-PCR primers	
survivin forward	ATG GGT GCC CCG ACG TTG
survivin reverse	AGA GGC CTC AAT CCA TGG
actin forward	ATC ATT GCT CCA CCA GAA CG
actin reverse	AAG GTA GAT AGA GAA GCC AAG

a The red portion is complementary with the red portion of probe S1, the blue portion is complementary with the blue portion of probe S2.
b The single mismatched base is highlighted in the box.
c The underlined region is the complementary sequence to the underlined region of probe S2.
d FITC is the abbreviation of fluorescein isothiocyanate.

Apparatus. Transmission electron micrographs were obtained on JEM-1011 and JEM-2100 transmission electron microscope (JEOL Ltd., Japan). Scanning electron micrographs were obtained on S-4800 scanning electron microscope（Hitachi, Japan). UV-vis absorption spectra

were recorded using a UV-vis spectrophotometer (Nanodrop-2000C, Nanodrop, USA). The ζ-potential was acquired with a Malvern (Nano-Z, Malvern Instruments Ltd., Britain) instrument. The cell viability assay was performed using a Thermo Scientific Varioskan Flash (Thermo Fisher Scientific, USA). Real-time quantitative, reverse transcription PCR was detected by Bio-Rad C1000 Real-Time PCR Detection System (Bio-Rad Laboratories, Inc, USA). The melting temperature experiment was performed on Epoch 2 Microplate Spectrophotometer (BioTek Instruments, Inc, USA).

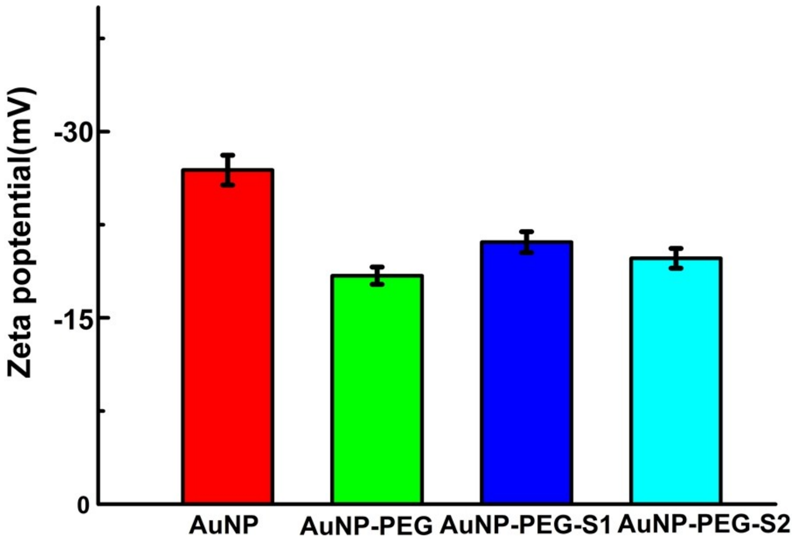

Fig. S1 Zeta potentials of AuNPs, AuNPs-PEG, AuNPs-PEG-S1 and AuNP-PEG-S2.

The asymmetrical modification process was characterized by Zeta potential analysis. The AuNPs exhibited a negative ζ potential of -26.9 mV, as this material was capped by tannic acid. The ζ potential of AuNPs-PEG was up to -18.4 mV (Fig. S1), the change was attributed to a large number of tannic acid displaced by SH-PEG. The obtained asymmetrically modified monomer probes (AuNPs-PEG-S1 and AuNPs-PEG-S2) showed slight decrease of ζ potential (-21.1 mV and -19.8 mV). The slight change was due to that only limited DNA probes with negative phosphate groups were modified on AuNPs-PEG after asymmetrical PEGylation.

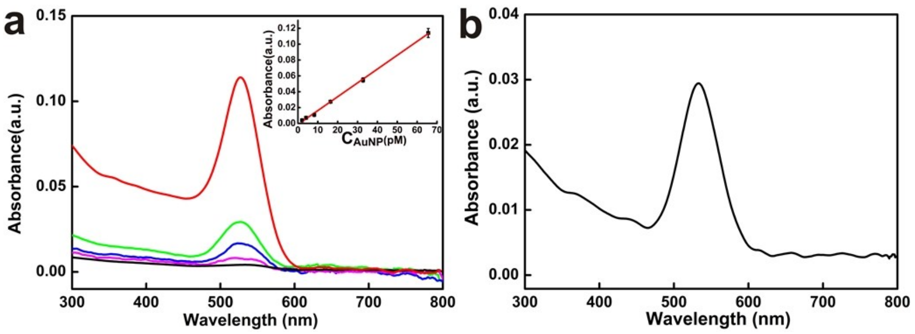

Fig. S2 (a) The absorbance intensities of different concentrations of AuNPs, the insert curve is the linear relationship between absorbance intensity and AuNPs concentrations. (b) The absorbance intensities of AuNPs-PEG after asymmetrical PEGylation.

The UV-Vis absorbance intensities of a series concentration of AuNPs were detected to establish a calibration curve for calculating the concentration of AuNPs-PEG after asymmetrical PEGylation. As shown in Fig. S2a, as the AuNPs concentration increasing, the intensity was enhanced, a linear range from 2.05 to 65.6 pM was observed with $I_A = -1.28 \times 10^{-3} + 1.75 \times 10^{-3} C_{AuNPs}$ (pM) (n = 6, R = 0.9993). After asymmetrical PEGylation process, the intensity of AuNPs-PEG was 0.0302 (Fig. S2b) and the concentration was 18 pM calculated by the above linear equation.

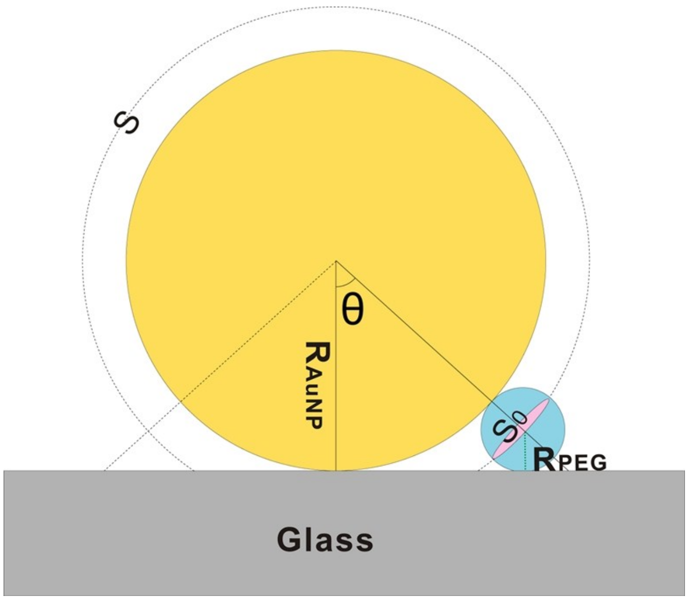

Fig. S3 The cross section of simulate structure composed by AuNP and PEG molecule in the asymmetrical modification.

The cross section of simulate structure composed by AuNP and PEG molecule in the

asymmetrical modification was shown in Fig. S3. The Flory radius for PEG molecule (M_W3000) was calculated by the followed equation[1]: $R_{F3} \approx a_m n_p^{3/5}$, a_m is the length of single oxyethylene monomer unit, n_p is the number of oxyethylene monomer unit. Based on the molecular formula and weight, n_p of PEG molecule used in this work is nearly 63. As the oxyethylene monomer unit of PEG[2]: $a_m \approx 0.35$ nm, R_{F3} of PEG molecule is roughly 4.20 nm (named as R_{PEG}, represented by green dotted line in Fig. S3) according to the above equation. Then during the asymmetrical modification, the area of each PEG molecule (named as S_0) occupied on nanoparticle is nearly 55.39 nm^2. Upon the radius of PEG (R_{PEG}), AuNP (R_{AuNP}) and the definition of cosine, we calculated the detailed value of θ (showed in Fig. S3) was 65.89 °. Based on the definition of solid angle Ω: $\Omega = 2\pi\ (1-\cos\theta)$, we calculated the solid angle of the asymmetrically modified AuNP: $\Omega = 4\pi\ R_{PEG} /(R_{AuNP} + R_{PEG})$. Hence, the total area of PEG modified on each AuNP monomer (named as S): $S = 4\pi\ (R_{AuNP} + R_{PEG})^2\ (1\text{-}\Omega/4\pi)$, the detail value is about 1783.52 nm^2. Then the number of PEG molecule (named as N_{PEG}) modified on each AuNP is approximate to the value of S / S0, at most 32 PEG molecule could be modified on each AuNP.

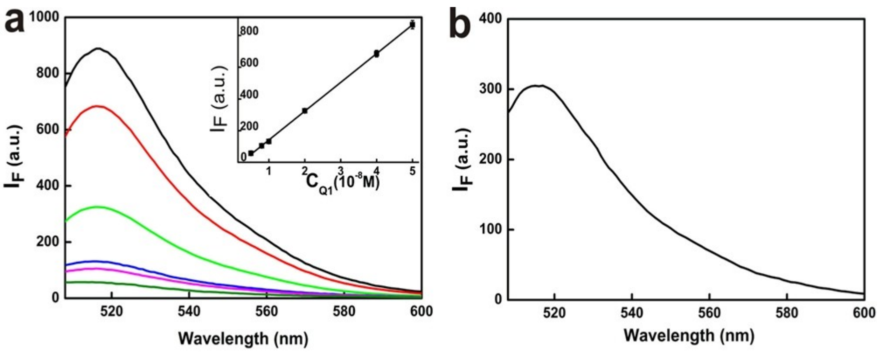

Fig. S4 (a) The fluorescence intensities of different concentrations of Q1, the insert curve is the linear relationship between intensity and Q1 concentrations. (b) The fluorescence intensities of Q1 retained in supernatant after asymmetrical modification.

For quantifying the oligonucleotides loaded on each AuNP monomer, the fluorescence intensity of series concentration oligonucleotides (Q1) labeled by FITC was detected to establish a calibration curve. As shown in Fig. S4a, as the concentration of Q1 increased, the intensity was enhanced, a linear range from 5.0×10^{-9} to 5.0×10^{-8} M was observed with $I_F = -39.67 + 181.37\ C_{Q1}\ (10^{-8}\ M)$ (R = 0.9997). After modification process, the mean intensity of Q1 retained in supernatant was 304.9

(Fig. S4b) and the concentration was 1.90 × 10^{-8} M calculated by the above linear equation. As the total amount of Q1 used in the process was 2.0 × 10^{-8} M, then the Q1 concentration loaded on AuNP monomer was estimated to be 0.10 × 10^{-8} M (named as $C_{DNA\text{-}below}$). The number of thiol oligonucleotides loaded on each AuNP monomer was 5 ±1 calculated by the followed equation: $N_{DNA} = N_{total\text{-}DNA} / N_{total\text{-}AuNP} = C_{DNA\text{-}below} \cdot Vsolution \cdot N_A / C_{AuNP} \cdot Vsolution \cdot N_A$.

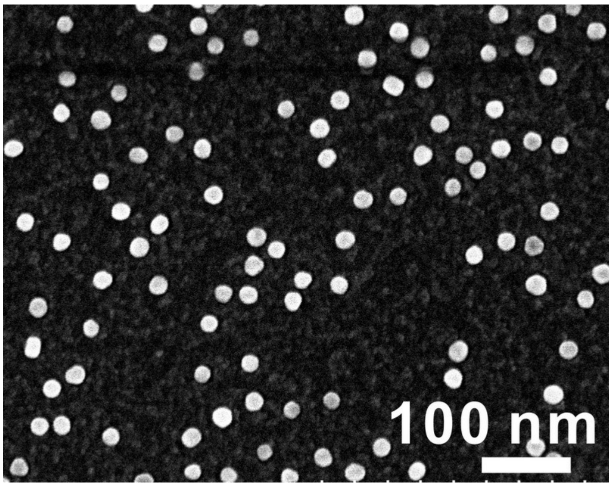

Fig. S5 SEM images of asymmetrically modified monomer probes.

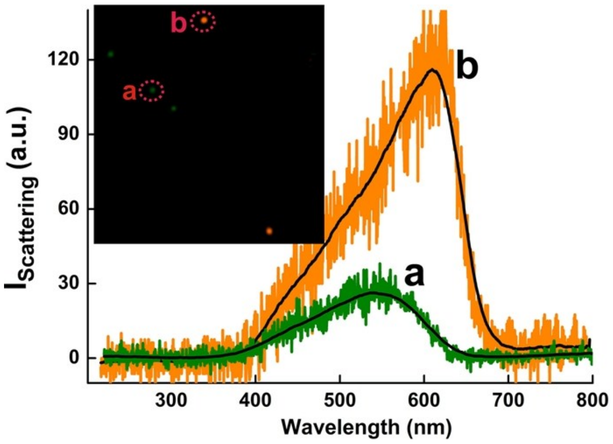

Fig. S6 The scattering spectra of single monomer probe (30 nm AuNPs was used, curve a) and dimer (curve b) in the contrast experiment, the inset is the dark-field image of the probes solution after reacted with target molecules (T1, 0.01pM).

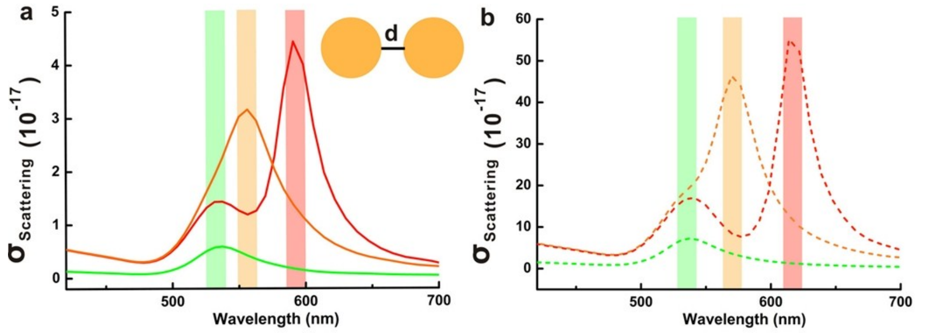

Fig. S7 Theoretically simulated spectra of single AuNP (green lines), AuNP dimer with different interparticle distance (d=3 nm, orange lines; d=1 nm, red lines) of diameter 20 nm (a) and 30 nm (b).

The finite-difference time domain (FDTD) method was used to simulate the LSPR properties of a monomer and dimer simulated. In order to match the experiment observation, single AuNP and two AuNPs with differernt interparticle distances were used as model for simulation. In the simulated structure, the refractive index of background was 1.33 and a total-field scattered-field source with circular polarization by averaging over two orthogonal polarizations was used for investigating the scattering efficiency from 300 to 800 nm (Fig. S7). The outcomes of FDTD confirmed no matter choosing 20 nm and 30 nm gold nanoparticles as models, the LSPR spectra of a dimer were distinctly red-shifted compared to that of a monomer, which was attributed to the strong interparticle coupling effect. When the interparticle distance decreased to 1 nm, the shift became dramatic and the scattering intensity increased sharply. Meanwhile, another peak at ~540 nm appeared, which was attributed to the strong interaction between single-particle multipoles when the particles nearly touching.[3] As theoretical simulation simplified the condition in experimental measurement, slight distinctions existed in the results of simulation and experiment is reasonable.[4] As reported, the simulation results based on relative big size nanoparticle were more accurate, which may attribute to increase of the portion of Mie scattering contributed to the total extinction along with the larger nanoparticle size.[5]

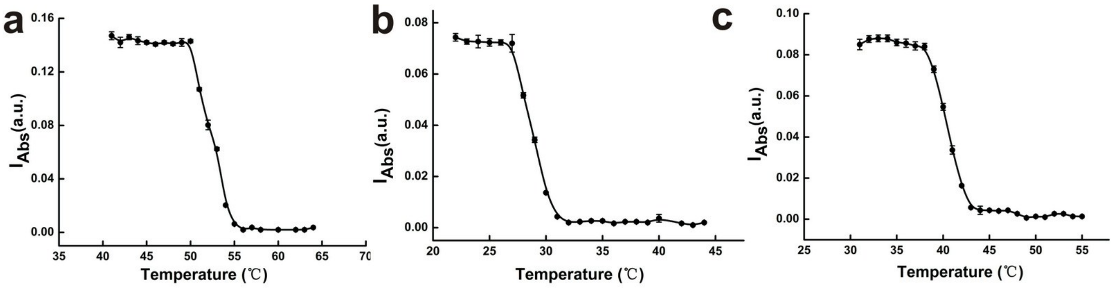

Fig. S8 The melting temperature assay of the reaction solution containing monomer probes with target (T1), single mismatched strand at middle (M-C1) or single mismatched strand at head (H-C1), respectively.

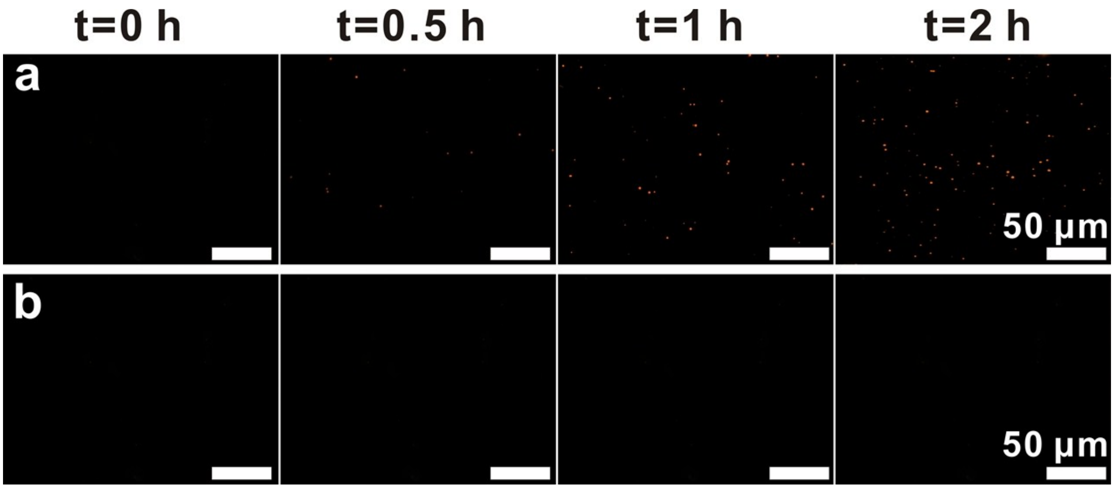

Fig. S9 Dark-field images of monomer probes incubated with target (T1, 0.1 pM, a) or control (M-C1, 0.1 pM, b) oligonucleotides at 37 °C for different time.

Dark-field assay was also used to study the selectivity of this plasmonic sensor. As shown in Fig. S9, at time t = 0 no light spot was detected, indicating the monomer probes were dispersive and a zero-background was successfully obtained. When incubated with T1 for a period of time, some orange spots, representing the dimers scattering under dark-field microscopy, were detected (Fig. S9a). With incubating time prolonging, more and more orange spot were displayed. Nevertheless, in the control experiment, no visible spot was observed even up to 2 h (Fig. S9b), demonstrating there was no hybridization reaction inducing the formation of dimers.

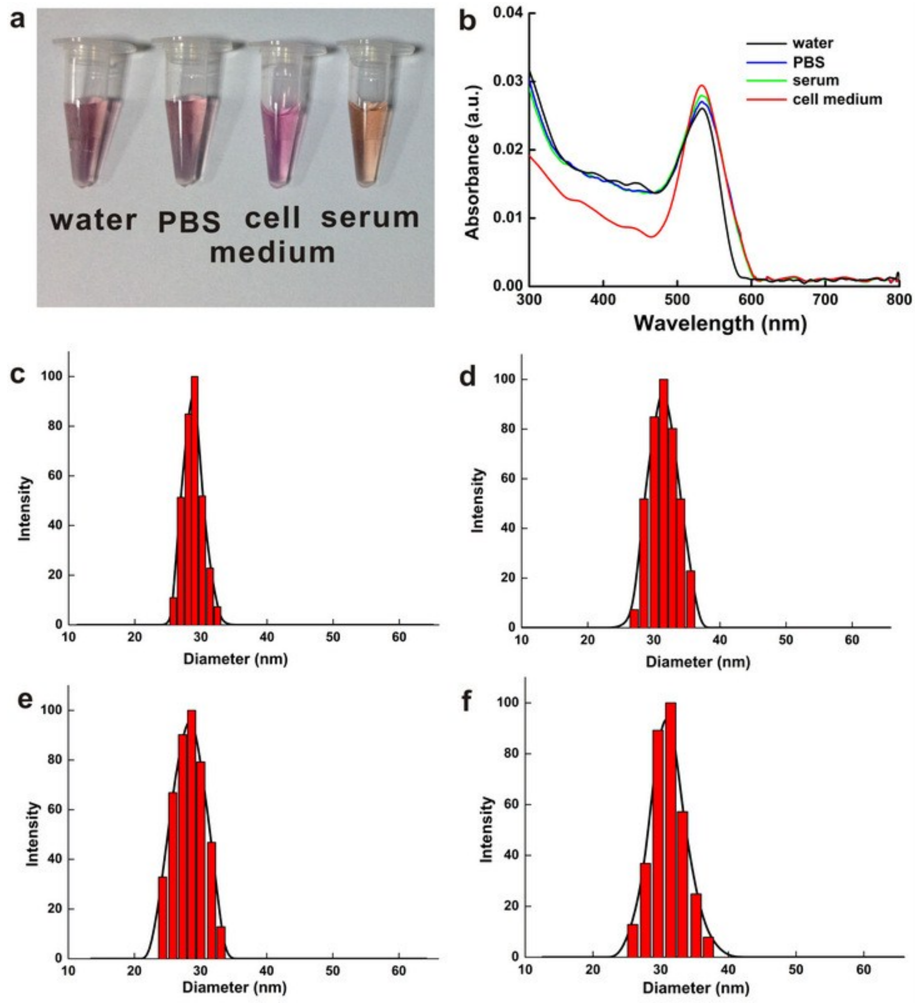

Fig. S10 (a) Asymmetrically modified monomer probes in different biological solutions- water, PBS buffer solution, cell medium and serum. (b) Optical absorption spectra of the monomer probes in different biological solutions. (c-f) DLS characterization of the monomer probes in water (c), PBS buffer solution (d), cell medium (e) and serum (f).

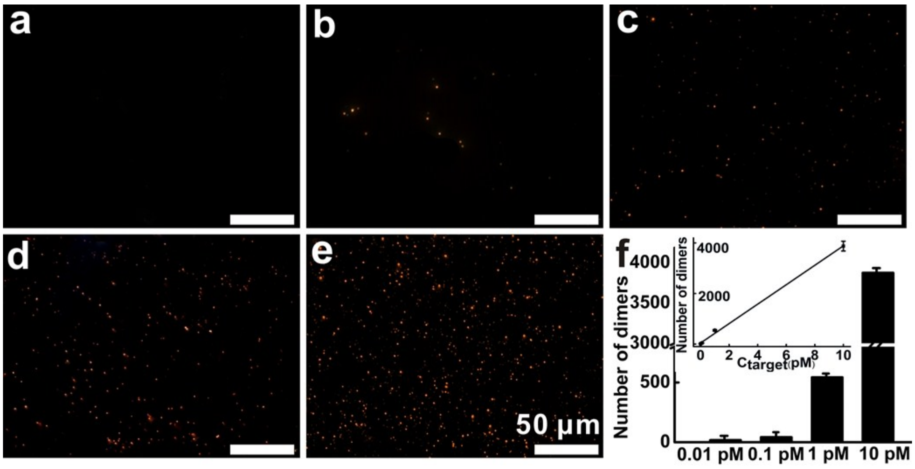

Fig. S11 Dark-field images of nanoparticles after addition of different concentration of target oligonucleotide (T1, from a to e) in Tris-buffer solution: 0, 0.01, 0.1, 1, 10 pM, each image area is 235 μm×176 μm. (f) The number of dimers at different concentration points. Inset is the linear relationship between number of dimers and T1 concentration.

In the presence of target, a nanoparticle-dimer was formed based on the hybridization of two monomer probes to a single target mRNA and the number of dimers was significantly increased with the target concentration increasing. At the concentration of 0.01 pM, the number of orange spots representing the scattering of the dimer was 17. While increasing the concentration to 10 pM, the number was greatly increased to 3875 (counted by the Image-Pro-Plus software), a linear range from 1.0×10^{-14} to 1.0×10^{-11} M was observed with $N = 41+385\ C_{DNA}$ (pM) ($R = 0.997$).

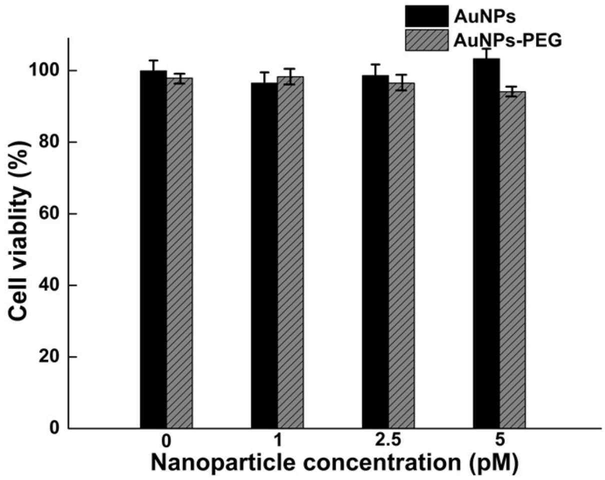

Fig. S12 Cytotoxicity assays of HeLa cells incubated with different concentrations of AuNPs and asymmetrically functionalized AuNPs-PEG.

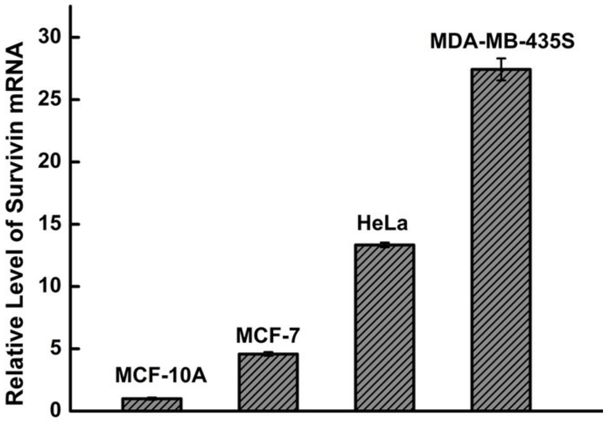

Fig. S13 qRT-PCR of survivn mRNA expression level in MCF-10A, MCF-7, HeLa and MDA-MB-435S cells, relative level of survivn mRNA was normalized to actin mRNA.

Reference:

1. D. Marsh, R. Bartucci and L. Sportelli, *BBA-Biomembranes*, 2003, **1615**, 33-59.
2. K. Hristova and D. Needham, *Macromolecules*, 1995, **28**, 991-1002.
3. I. Romero, J. Aizpurua, G. W. Bryant and F. J. Garcia de Abajo, *Opt. Express*, 2006, **14**, 9988-9999.
4. X. Lan, Z. Chen, B.-J. Liu, B. Ren, J. Henzie and Q. Wang, *Small*, 2013, **9**, 2308-2315.
5. P. K. Jain, X. Huang, I. H. El-Sayed and M. A. El-Sayad, *Plasmonics*, 2007, **2**, 107-118.
